# Add-On Effect of Honeysuckle in the Treatment of Coronavirus Disease 2019: A Systematic Review and Meta-Analysis

**DOI:** 10.3389/fphar.2021.708636

**Published:** 2021-09-15

**Authors:** Xu-Qin Du, Li-Peng Shi, Wen-Fu Cao, Zhi-Wei Chen, Biao Zuo, Jin-Yuan Hu

**Affiliations:** ^1^ College of Traditional Chinese Medicine, Chongqing Medical University, Chongqing, China; ^2^ Chongqing Key Laboratory of Traditional Chinese Medicine for Prevention and Cure of Metabolic Diseases, Chongqing, China; ^3^ Department of Chinese Traditional Medicine, The First Affiliated Hospital of Chongqing Medical University, Chongqing, China

**Keywords:** coronavirus disease 2019, honeysuckle, systematic review, meta-analysis, randomized controlled trials

## Abstract

**Background:** The outbreak of coronavirus disease 2019 (COVID-19) has rapidly spread to become a global emergency since December 2019. Chinese herbal medicine plays an important role in the treatment of COVID-19. Chinese herbal medicine honeysuckle is an extremely used traditional edible and medicinal herb. Many trials suggest that honeysuckle has obtained a good curative effect for COVID-19; however, no systematic evaluation on the clinical efficacy of honeysuckle in the treatment of COVID-19 is reported. This study aimed to evaluate the efficacy and safety of Chinese herbal medicine honeysuckle in the treatment of COVID-19.

**Methods:** Seven electronic databases (PubMed, EMBASE, Cochrane Library, China National Knowledge Infrastructure, China Science and Technology Journal Database, Wanfang Database, and China Biology Medicine) were searched to identify randomized controlled trials (RCTs) of honeysuckle for adult patients (aged ≥ 18 years) with COVID-19. The Cochrane Risk of Bias Tool was applied to assess the methodological quality of trials. Review Manager 5.3 software was used for data analysis.

**Results:** Overall, nine RCTs involving 1,286 patients were enrolled. Our meta-analyses found that combination therapy of honeysuckle and conventional therapy was more effective than conventional therapy alone in lung computed tomography (CT) [relative risk (RR) = 1.24, 95% confidence interval (95%CI) (1.12, 1.37), *P* < 0.0001], clinical cure rate [RR = 1.21, 95%CI (1.12, 1.31), *P* < 0.00001], and rate of conversion to severe cases [RR = 0.50, 95%CI (0.33, 0.76), *P* = 0.001]. Besides, combination therapy can improve the symptom score of fever, cough reduction rate, symptom score of cough, and inflammatory biomarkers (white blood cell (WBC) count; C-reactive protein (CRP)) (*P* < 0.05).

**Conclusion:** Honeysuckle combined with conventional therapy may be beneficial for the treatment of COVID-19 in improving lung CT, clinical cure rate, clinical symptoms, and laboratory indicators and reducing the rate of conversion to severe cases. Besides, combination therapy did not increase adverse drug events. More high-quality RCTs are needed in the future.

## Introduction

Since December 2019, coronavirus disease 2019 (COVID-19) caused by severe acute respiratory syndrome coronavirus 2 (SARS-CoV-2) has become a public health emergency of global concern (Sattler et al., 2020). As of May 6, 2021, more than 154.815 million confirmed cases and more than 3.236 million deaths had been reported globally (World Health Organization (WHO), 2021). Thus, there is an urgent need to prevent and treat COVID-19.

Through a series of prevention and medical treatment measures, the COVID-19 epidemic in China has been effectively controlled by May 6, 2021, with 103,731 confirmed cases and 98,392 cured cases (World Health Organization (WHO), 2021; National Health Commission of the people’s Republic of China, 2021). Chinese herbal medicine plays an important role in the treatment of COVID-19 in view of no specific drugs approved for COVID-19. Chinese herbal medicine honeysuckle is an extremely used traditional edible-medicinal herb (Li et al., 2021). Pharmacological studies have already proved honeysuckle’s ideal clinical therapeutic effects on inflammation and infectious diseases (Li et al., 2021). Also, it is reported that honeysuckle can effectively alleviate clinical symptoms of COVID-19 (Hu et al., 2020; Zhang et al., 2020) and inhibit SARS-CoV-2 replication (Zhou et al., 2020).

At present, there are only few trials on the treatment of COVID-19 with honeysuckle (Hu et al., 2020; Zhang et al., 2020), but many trials on the treatment of COVID-19 used Chinese herbal medicine including honeysuckle as the main components (Ai et al., 2020; Ding et al., 2020; Duan et al., 2020). These trials suggest that honeysuckle has obtained a good curative effect for COVID-19 (Hu et al., 2020; Zhang et al., 2020). Presently, there is no systematic evaluation report on the clinical efficacy of honeysuckle in the treatment of COVID-19. This review aimed to critically evaluate the effectiveness and safety of honeysuckle for COVID-19.

## Methods

The preferred reporting item for systematic review and meta-analysis (PRISMA) Evaluation Scale was used for reporting the results of this review (Moher et al., 2009). The protocol for this review is available in PROSPERO (https://www.crd.york.ac.uk/prospero/, registration number is CRD42020224312).

### Database and Search Strategies

The following seven databases were retrieved, including PubMed, EMBASE, Cochrane Library, China National Knowledge Infrastructure (CNKI), China Science and Technology Journal Database (VIP), Wanfang Database, and China Biology Medicine (CBM), from December 2019 to May 2021. There was no language restriction. The grouped keywords used as search strategy were as follows: (“traditional Chinese medicine” OR “Chinese herbal medicine” OR “honeysuckle” OR “lonicera” OR “jinyinhua”) AND (“coronavirus disease 2019” OR “COVID-19” OR “novel coronavirus pneumonia” OR “SARS-CoV-2”) AND (“clinical trial” OR “randomized controlled trial” OR “randomised controlled trial” OR “lin chuang yan jiu”). The grouped keywords could be modified according to different databases.

Potentially eligible trials were obtained by manually searching the reference lists of published reviews and meta-analyses. We also retrieved the unpublished papers on honeysuckle for COVID-19.

### Inclusion and Exclusion Criteria

We considered the following inclusion criteria: 1) study design: randomized controlled trials (RCTs); 2) participants: adult patients (aged ≥ 18 years) diagnosed with COVID-19; the diagnostic criteria of COVID-19 refer to “ Diagnosis and Treatment Guideline for COVID-19 (Trial 8th Edition) ” (National Health Commission of the people’s Republic of China, 2020); 3) interventions: patients in the treatment group were treated with honeysuckle alone or a combination treatment of honeysuckle and controls; the dose of honeysuckle was 5–30 g, along with a duration range of 5–15 days; the form and dosage of honeysuckle were included in the study description; 4) control: patients in the control group were treated by any conventional therapy or placebo; 5) outcomes: lung computed tomography (CT) was the primary outcome. High-resolution CT was used to observe changes in the lung field before and after treatment. The secondary outcomes included clinical cure rate, viral nucleic acid testing, rate of conversion to severe cases, clinical symptoms (e.g., fever, cough, and fatigue), inflammatory biomarkers [e.g., white blood cell (WBC) count, lymphocyte (LYM) count, and C-reactive protein (CRP)], and adverse drug events (e.g., adverse events rate, diarrhea, and liver damage).

We considered the following exclusion criteria: 1) study design: non-RCTs, such as retrospective studies, observational studies, case reports, and cross-over studies; non-RCTs were excluded due to potential high risk of bias and confounding; 2) participants: patients with a suspected diagnosis of COVID-19; 3) repeated data studies; 4) reviews.

Two reviewers independently screened the trials from seven databases according to the eligibility criteria. Duplicate publications were removed. Through reading the title, abstract, and full text, reviewers excluded the non-RCTs and irrelevant trials. Two reviewers independently extracted data according to the eligibility criteria. Any disagreements were consulted by a third reviewer.

### Data Collection

The following information was documented in the data extraction tables: basic characteristics (e.g., the title, first authors’ name, publication date, intervention schedule of treatment and control groups, and treatment duration), participant characteristics (e.g., age, gender, and sample size), outcome measures, and adverse drug events.

### Quality Assessment

The methodological quality was independently evaluated by two reviewers according to the Cochrane Collaboration’s tool (Higgins and Green, 2014). There were seven items of risk of bias (ROB): random sequence generation, allocation concealment, blinding of participants and personnel, blinding of outcome assessment, incomplete outcome data, selective reporting, and other biases. Each item was assessed at low ROB, high ROB, or unclear ROB. Any disagreements between reviewers were resolved by consultation with a third reviewer.

### Statistical Analysis

Review Manager 5.3 software (the Cochrane Collaboration, 2014) was used to perform the quantitative synthesis. Relative risk (RR) was used for the dichotomous variables. Mean difference (MD) or standard mean difference (SMD) was used for the continuous variables. Confidence intervals (CIs) were set as 95%. Heterogeneity was tested with the χ2 test and the *I* ^*2*^ statistical value. Assuming that the *p*-value from the χ2 test was more than 0.10 or *I* ^*2*^ ≤ 50%, a fixed-effect model was used to assess the differences between two groups; otherwise, a random-effects model was applied. A subgroup analysis of the primary outcome was performed according to the clinical classification of COVID-19. Subgroup analyses of viral nucleic acid testing and rate of conversion to severe cases were performed according to the Chinese herbal medicine component. Sensitivity analysis was conducted by the leave-one-out method (Panahi et al., 2015). When the number of trials on an outcome measure was larger than ten, a funnel plot analysis was performed to evaluate the reporting bias (Liu et al., 2020). Statistically significant results were defined by *p* < 0.05.

## Results

### Study Selection

**FIGURE 1:**
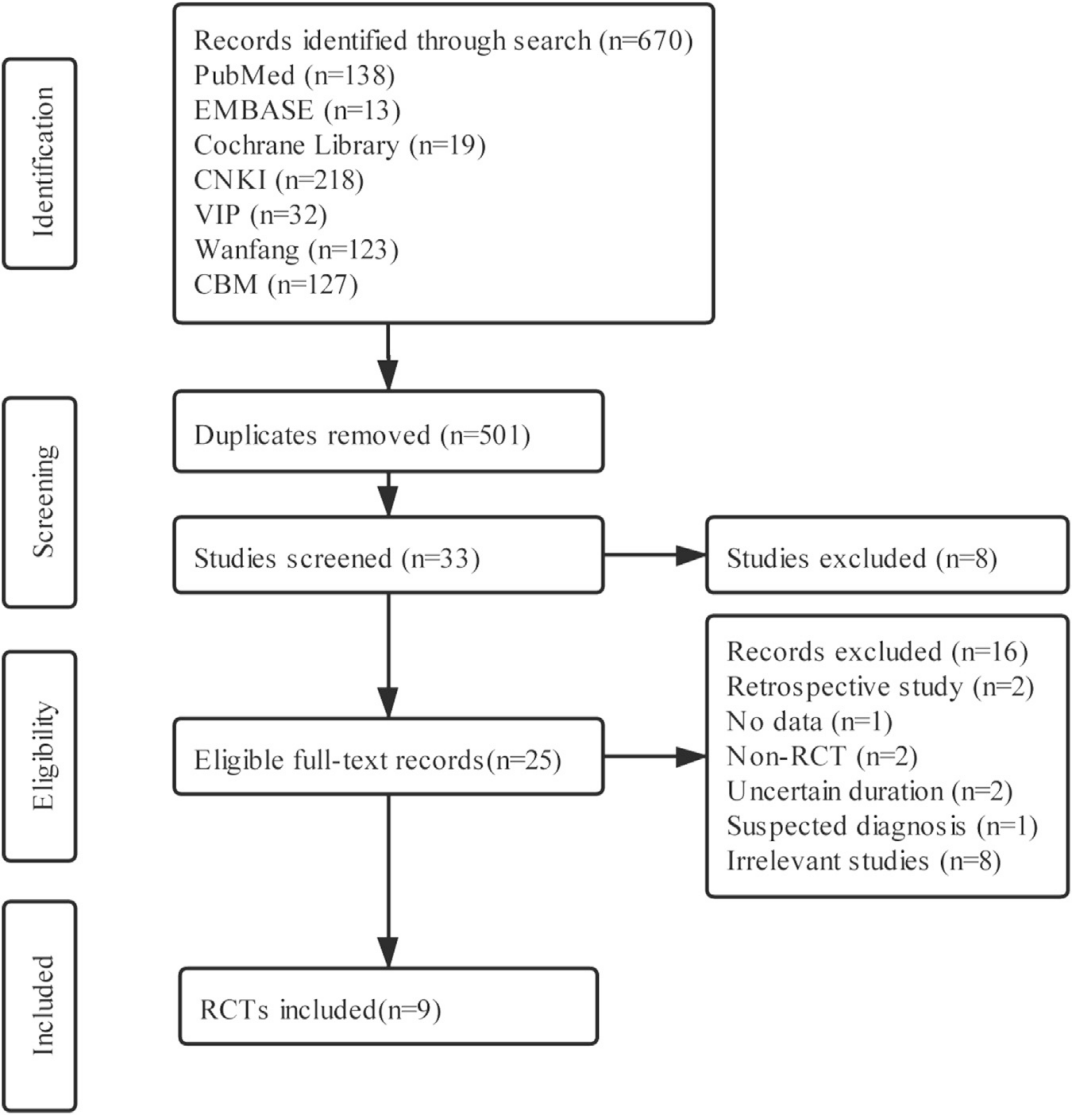
The flowchart of study selection.

The flowchart of study selection is shown in (Figure 1). A total of nine eligible trials were included (Fu et al., 2020a; Ai et al., 2020; Ding et al., 2020; Duan et al., 2020; Fu et al., 2020b; Hu et al., 2020; Yu et al., 2020; Zhang et al., 2020; Hu et al., 2021). One article was published online in English (Hu et al., 2021), and the rest were reported online in Chinese.

### Study Characteristics

The characteristics of included trials are listed in Table 1. All the trials were conducted in China in 2020, among them, two studies were multicentered trials (Hu et al., 2020; Hu et al., 2021) and seven were single-centered trials (Ai et al., 2020; Ding et al., 2020; Duan et al., 2020; Hu et al., 2020; Yu et al., 2020; Zhang et al., 2020; Hu et al., 2021). The sample size of the included trials ranged from 65 to 295 (total 1,286). The treatment duration ranged from 5 to 15 days. Patients in the treatment group were treated with combination therapy of honeysuckle and controls. Control groups used conventional therapy. In each trial, conventional therapy in the treatment group was identical to the control group. Two trial intervention groups were Chinese medicine compound drugs (Pneumonia No. 1 formula and Qingfei Touxie Fuzheng recipe) (Ai et al., 2020; Ding et al., 2020). And the other trials were Chinese patent medicine (Duan et al., 2020; Fu et al., 2020a; Fu et al., 2020b; Hu et al., 2020; Yu et al., 2020; Zhang et al., 2020; Hu et al., 2021). Conventional therapy included oxygen therapy, drugs, and symptomatic therapies. The drugs used in the control group were arbidol, lopinavir, interferon-α, and ribavirin. Two trial control groups did not provide specific therapy medicine (Duan et al., 2020; Hu et al., 2021).

**TABLE 1:** The characteristics of included trials.

First author	Type of COVID-19	Sample size (M/F)	Age (yrs)	Intervention	Control	Duration	Outcome measures
Ai et al. (2020)	Nonsevere	T:55 (24/31) C:43 (17/26)	T:57.4 ± 21.3 C:50.8 ± 23.5	Pneumonia No.1 formula (1 dose/d) + conventional therapy	Conventional therapy including arbidol, lopinavir/tonavir, chloroquine, and symptomatic treatment	12 days	Clinical cure rate, inflammatory biomarkers, and adverse events
Ding et al. (2020)	Mild/moderate/severe	T:51 (39/12) C:49 (39/10)	T:57.4 ± 21.3 C:50.8 ± 23.5	Qingfei Touxie Fuzheng recipe (1 dose/d) + conventional therapy	Conventional therapy including interferon-α, ribavirin, quinolones and/or third-generation cephalosporins, and symptomatic treatment	10 days	Lung CT, clinical symptoms, inflammatory biomarkers, and adverse events
Duan et al. (2020)	Mild	T:82 (39/43) C:41 (23/18)	T:51.99 ± 13.88 C:50.29 ± 13.17	Jinhua Qinggan granule (1 dose/time, tid) + conventional therapy	Conventional therapy including antiviral, anti-infection, and other symptomatic treatments	5 days	Clinical symptoms and adverse events
Fu et al. (2020a)	Mild/moderate	T:32 (17/15) C:33 (19/14)	T:43.26 ± 7.15 C:43.68 ± 6.45	Toujie Quwen granule (1 dose/time, bid) + conventional therapy	Conventional therapy including abidor tablets, moxifloxacin tablets, and ambroxol tablets	10 days	Lung CT, clinical cure rate, rate of conversion to severe cases, clinical symptoms, inflammatory biomarkers, and adverse events
Fu et al. (2020b)	Moderate	T:37 (19/18) C:36 (19/17)	T:45.26 ± 7.25 C:44.68 ± 7.45	Toujie Quwen granule (1 dose/time, bid) + conventional therapy	Conventional therapy including abidor tablets and ambroxol tablets	15 days	Clinical cure rate, rate of conversion to severe cases, clinical symptoms, inflammatory biomarkers, and adverse events
Hu et al. (2020)	Moderate	T:100 (49/51) C:100 (55/45)	T:47.00 ± 14.06 C:49.28 ± 11.14	Jinyinhua oral liquid (120 ml/time, tid) + conventional therapy	Conventional therapy including interferon-α, lopinavir/tonavir tablets, and symptomatic treatment	10 days	Lung CT, virus nucleic acid testing, rate of conversion to severe cases, and adverse events
Hu et al. (2020)	Mild/moderate	T:142 (79/63) C:142 (71/71)	T:50.4 ± 15.2 C:51.8 ± 14.8	Lianhua Qingwen capsule (6 g, tid) + conventional therapy	Conventional therapy including oxygen therapy, antiviral medications, and symptomatic therapies	14 days	Lung CT, clinical cure rate, virus nucleic acid testing, rate of conversion to severe cases, clinical symptoms, and adverse events
Yu et al. (2020)	Mild/moderate	T:147 (82/65) C:148 (89/59)	T:48.27 ± 9.56 C:47.25 ± 8.67	Lianhua Qingwen granule (6 g, tid) + conventional therapy	Conventional therapy including abidor tablets, moxifloxacin tablets, and ambroxol tablets	7 days	Lung CT, clinical cure rate, rate of conversion to severe cases, clinical symptoms inflammatory biomarkers, and adverse events
Zhang et al. (2020)	Moderate	T:80 (50/30) C:40 (23/17)	T:53.4 ± 13.70 C:52.0 ± 14.10	Jinyinhua oral liquid (60 ml/time, tid) + conventional therapy	Conventional therapy including interferon-α, lopinavir, tonavir tablets, and symptomatic treatment	10 days	Rate of conversion to severe cases, clinical symptoms, and adverse events

### Description of Honeysuckle

The description of honeysuckle in each trial is shown in Table 2. Honeysuckle was used in the dosage formulations of granules (55.55%) (Ai et al., 2020; Duan et al., 2020; Fu et al., 2020a; Fu et al., 2020b; Yu et al., 2020), decoction (11.11%) (Ding et al., 2020), oral liquid (22.22%) (Hu et al., 2020; Zhang et al., 2020), and capsule (11.11%) (Hu et al., 2021). The component of oral liquid (Hu et al., 2020; Zhang et al., 2020) is only honeysuckle. Honeysuckle is one component of Chinese herbal medicine in other dosage formulations.

**TABLE 2:** The description of honeysuckle in each trial.

References	Honeysuckle and Chinese herbal medicine	Components
Ai et al. (2020)	Pneumonia No.1 formula (granule)	Honeysuckle 15 g, Lianqiao 30 g, Qingdao 10 g, Huangqi 45 g, Shancigu 20 g, Huangqin 10 g, Daqingye 10 g, Chaihu 5 g, Chantui 10 g, Qianhu 5 g, Chuanbeimu 10 g, Zhebeimu 10 g, Wumei 30 g, Xuanshen 10 g, Fuling 30 g, and Taizishen 15 g
Ding et al. (2020)	Qingfei Touxie Fuzheng recipe (decoction)	Honeysuckle 30 g, Lianqiao 15 g, Mahuang 6 g, Shigao 20 g, Kuxingren 10 g, Lugen 30 g, Yiyiren 30 g, Jiangcan 10 g, Chantui 10 g, Huzhang 15 g, Jianghuang 10 g, Baishaoyao 10 g, Taizishen 20 g, and Gancao 15 g
Duan et al. (2020)	Jinhua Qinggan granule	Honeysuckle 10 g, Shigao 10 g, Mahuang 10 g, Kuxingren 10 g, Huangqin 10 g, Lianqiao 10 g, Zhebeimu 10 g, Zhimu 10 g, Niubangzi 10 g, Qinghao 10 g, Bohe 10 g, and Gancao10 g
Fu et al. (2020a)	Toujie Quwen granule	Honeysuckle 15 g, Lianqiao 30 g, Shancigu 20 g, Huangqin 10 g, Daqingye 10 g, Chaihu 5 g, Qinghao 10 g, Chantui 10 g, Qianhu 5 g, Chuanbeimu 10 g, Zhebeimu 10 g, Wumei 30 g, Xuanshen 10 g, Huangqi 45 g, Fuling 30 g, and Taizishen 15 g
Fu et al. (2020b)	Toujie Quwen granule	Honeysuckle 15 g, Lianqiao 30 g, Shancigu 20 g, Huangqin 10 g, Daqingye 10 g, Chaihu 5 g, Qinghao 10 g, Chantui 10 g, Qianhu 5 g, Chuanbeimu 10 g, Zhebeimu 10 g, Wumei 30 g, Xuanshen 10 g, Huangqi 45 g, Fuling 30 g, and Taizishen 15 g
Hu et al. (2020)	Jinyinhua oral liquid	Honeysuckle 10.8 g
Hu et al. (2020)	Lianhua Qingwen capsule	Honeysuckle, Lianqiao, Mahuang, Kuxingren, Shigao, Banlangen, Mianma, Guanzhong, Yuxingcao, Huoxiang, Dahuang, Hongjingtian, menthol, and Gancao
Yu et al. (2020)	Lianhua Qingwen granule	Honeysuckle, Lianqiao, Mahuang, Kuxingren, Shigao, Banlangen, Mianma, Guanzhong, Yuxingcao, Huoxiang, Dahuang, Hongjingtian, menthol, and Gancao
Zhang et al. (2020)	Jinyinhua oral liquid	Honeysuckle 5.4 g

## Methodological Quality

The results of the ROB assessment are shown in Table 3. In general, there was insufficient information available in all trials included in this study. The risks of bias of included trials were mostly “unclear risk.”

**TABLE 3:** Risk of bias.

References	A	B	C	D	E	F	G
Ai et al. (2020)	L	U	U	U	L	U	U
Ding et al. (2020)	L	U	U	U	L	U	U
Duan et al. (2020)	L	U	U	U	L	U	U
Fu et al. (2020a)	L	U	U	U	L	U	U
Fu et al. (2020b)	L	U	U	U	L	U	U
Hu et al. (2020)	L	U	U	U	L	U	U
Hu et al. (2020)	L	U	U	L	L	U	U
Yu et al. (2020)	L	U	U	U	L	U	U
Zhang et al. (2020)	U	U	U	U	L	U	U

A, random sequence generation (selection bias); B, allocation concealment (selection bias); C, blinding of participants and personnel (performance bias); D, blinding of outcome assessment (detection bias); E, incomplete outcome data (attrition bias); F, selective reporting (reporting bias); G, other biases; L, low risk; H, high risk; U, unclear.

### Results of the Meta-Analysis

**FIGURE 2:**
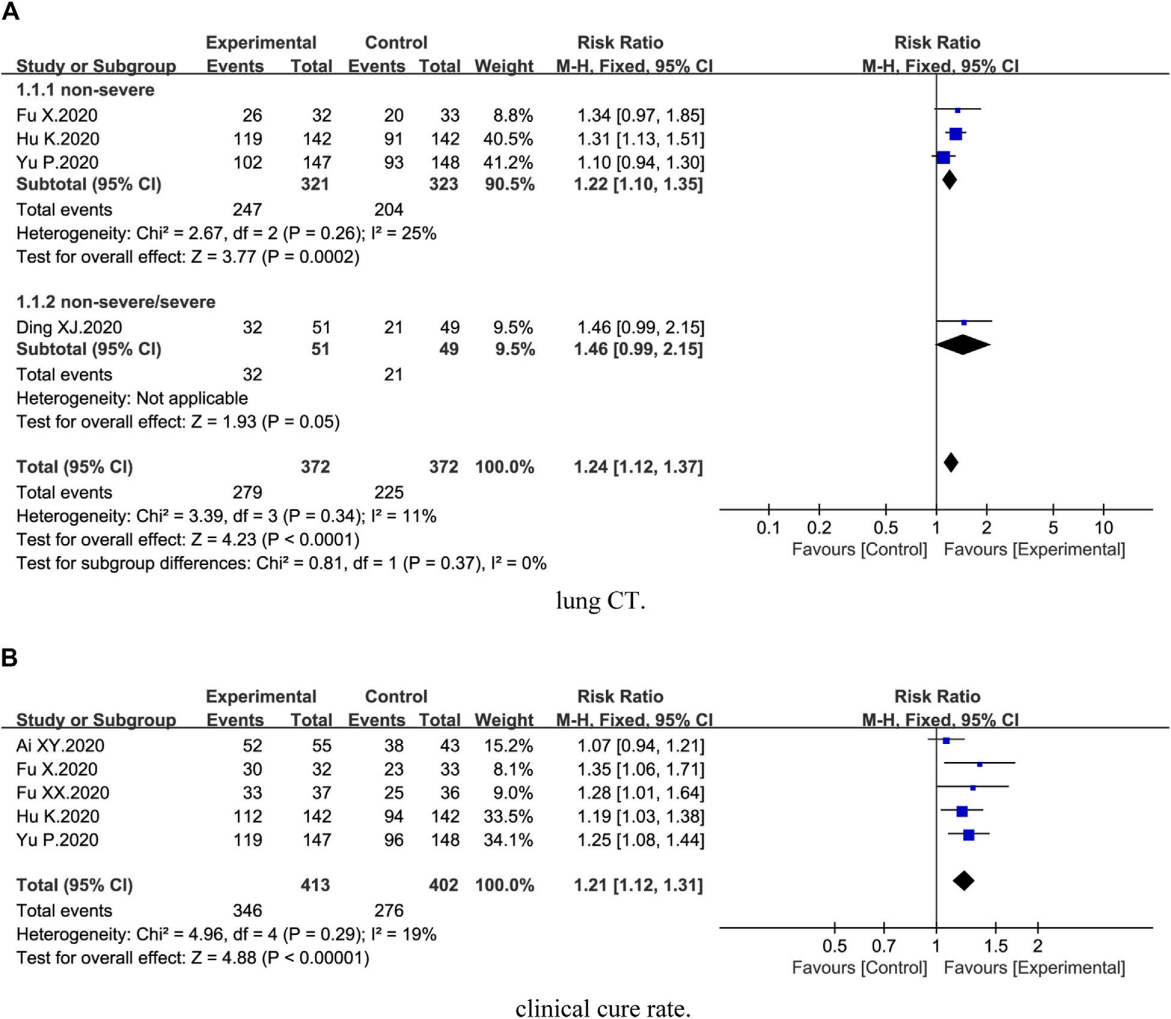
Forest plot of the effects of combination therapy for outcomes of **(A)** lung CT and **(B)** clinical cure rate.

Four trials (Ding et al., 2020; Duan et al., 2020; Hu et al., 2020; Yu et al., 2020) reported lung CT. Compared with conventional therapy alone, combination therapy of honeysuckle and conventional therapy exhibited a significant improvement on lung CT [4 trials, *n* = 744, RR = 1.24, 95%CI (1.12, 1.37), *I* ^*2*^ = 11%, *p* < 0.0001] (Figure 2A). Subgroup analysis revealed that combination therapy could better improve the lung CT of nonsevere COVID-19 [3 trials, *n* = 644, RR = 1.22, 95%CI (1.10, 1.35), *I* ^*2*^ = 25%, *p* = 0.0002] (Figure 2A).

Five trials (Ai et al., 2020; Fu et al., 2020a; Fu et al., 2020b; Yu et al., 2020; Hu et al., 2021) demonstrated the clinical cure rate. The results showed that clinical cure rate in the combination treatment groups was higher than the control groups [5 trials, *n* = 815, RR = 1.21, 95%CI (1.12, 1.31), *I* ^*2*^ = 19%, *p* < 0.00001] (Figure 2B).

**FIGURE 3:**
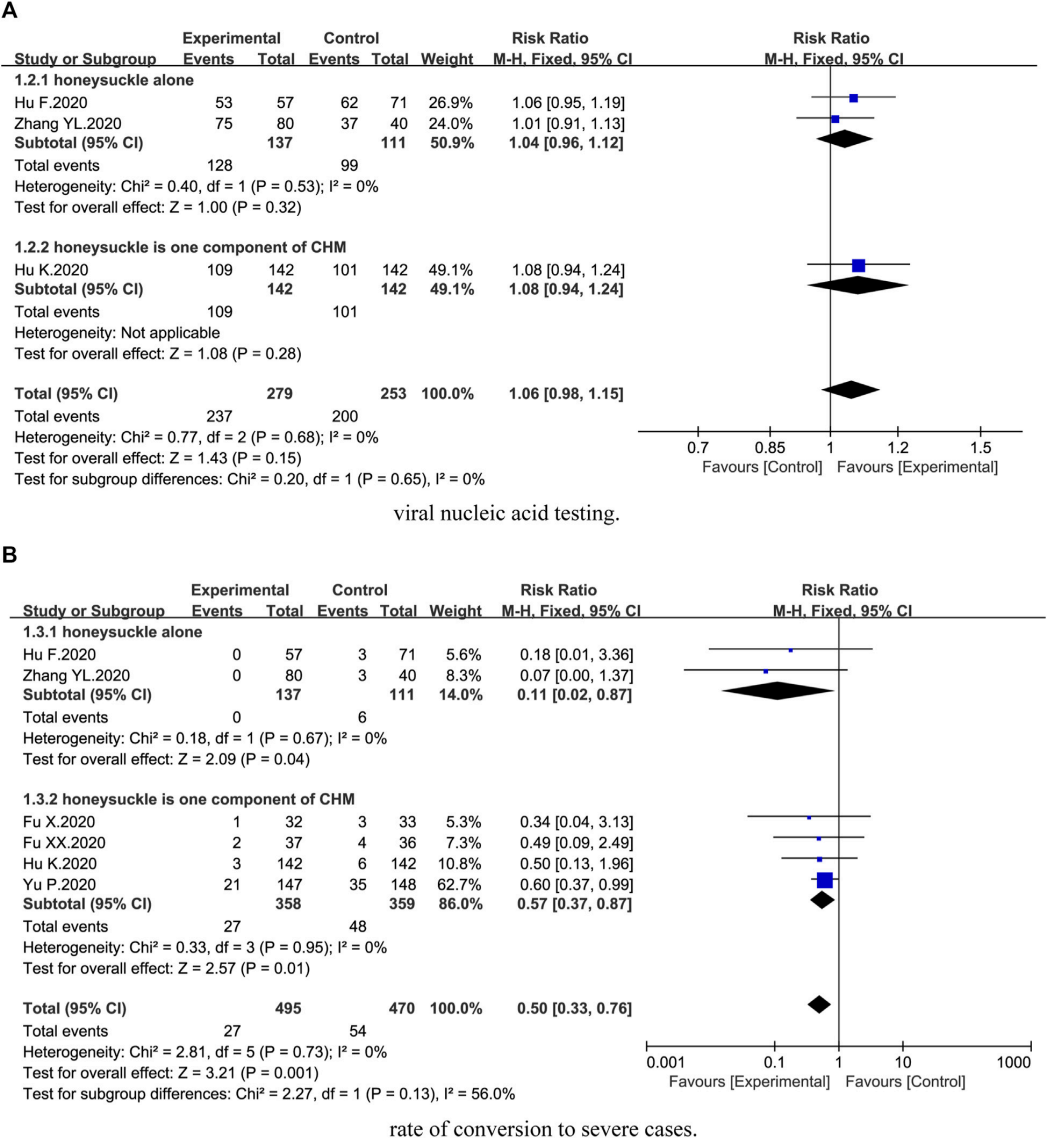
Forest plot of the effects of combination therapy for outcomes of **(A)** viral nucleic acid testing and **(B)** rate of conversion to severe cases.

Three trials (Hu et al., 2020; Zhang et al., 2020; Hu et al., 2021) described viral nucleic acid testing. Compared with the control groups, no statistical difference on viral nucleic acid testing was identified [3 trials, *n* = 532, RR = 1.06, 95%CI (0.98, 1.15), *I* ^*2*^ = 0%, *p* = 0.15] (Figure 3A). Subgroup analysis suggested no statistical difference between honeysuckle alone (*p* = 0.32) and Chinese herbal medicine formula (*p* = 0.28) (Figure 3A).

Six trials (Fu et al., 2020a; Fu et al., 2020b; Hu et al., 2020; Yu et al., 2020; Zhang et al., 2020; Hu et al., 2021) reported rate of conversion to severe cases. We found that combination therapy could significantly reduce the rate of conversion to severe cases [6 trials, *n* = 965, RR = 0.50, 95%CI (0.33, 0.76), *I* ^*2*^ = 0%, *p* = 0.001] (Figure 3B). Subgroup analysis showed that there was a significant difference between honeysuckle alone (*p* = 0.04) and Chinese herbal medicine formula (*p* = 0.01) (Figure 3B).

Six trials (Ding et al., 2020; Duan et al., 2020; Fu et al., 2020a; Fu et al., 2020b; Yu et al., 2020; Zhang et al., 2020) described clinical symptoms of fever, cough, and fatigue. Meta-analyses revealed that combination therapy could better improve the symptoms reduction rate and symptom score than conventional therapy (Table 4). As shown in Table 4, combination therapy could significantly improve the symptom score of fever, cough reduction rate, symptom score of cough, and symptom score of fatigue (*p* < 0.05). However, there was no significant difference in fever reduction rate and fatigue reduction rate between the combination treatment and control groups (*p* > 0.05).

**TABLE 4:** Comparison of clinical symptoms between the treatment group and control group.

Outcome measure	No. of trials	Samples			Statistical method	Effect estimate	*p*-value
		Total	Events/intervention	Events/control			
Fever cases	3	296	171/185	90/111	RR (random) 95%CI	0.11 (−0.10, 0.33)	0.31
Cough cases	3	260	135/167	56/93	RR (fixed) 95%CI	1.37 (1.15, 1.65)	0.0006
Fatigue cases	2	163	98/116	32/47	RR (random) 95%CI	1.20 (0.85, 1.69)	0.3
Symptom score of fever	3	433	—	—	RR (random) 95%CI	−0.62 (−0.79, −0.45)	< 0.00001
symptom score of cough	3	433	—	—	RR (random) 95%CI	−1.18 (−1.34, −1.03)	< 0.00001
Symptom score of fatigue	3	433	—	—	RR (random) 95%CI	−0.60 (−1.04, −0.17)	0.007

Five trials (Ai et al., 2020; Ding et al., 2020; Fu et al., 2020a; Fu et al., 2020b; Yu et al., 2020) reported inflammatory biomarkers. We found that combination therapy was beneficial for WBC count [3 trials, *n* = 433, MD = 0.38, 95%CI (0.31, 0.44), *I* ^*2*^ = 22%, *p* < 0.00001], LYM count [4 trials, *n* = 531, MD = 0.23, 95%CI (0.05, 0.41), *I* ^*2*^ = 97%, *p* = 0.01], and CRP level [4 trials, *n* = 533, MD = −12.95, 95%CI (−21.18, −4.01), *I* ^*2*^ = 98%, *p* = 0.004] to return to normal. And these differences were statistically significant (*p* < 0.05) (Table 5).

**TABLE 5:** Comparison of inflammatory biomarkers between the treatment group and control group.

Outcome measure	No. of trials	Samples	Statistical method	Effect estimate	*p*-value
WBC count	3	433	MD (fixed) 95%CI	0.38(0.31, 0.44)	< 0.00001
LYM count	4	531	MD (random) 95%CI	0.23(0.05, 0.41)	0.01
CRP	4	533	MD (random) 95%CI	−12.95(−21.18, −4.01)	0.004

All included trials reported adverse drug events. The common adverse drug events of combination therapy were nausea and vomiting, inappetence, diarrhea, headache, renal dysfunction, and abnormal liver function. As shown in Table 6, there was no significant difference in adverse events rate, diarrhea, and abnormal liver function between the combination treatment and control groups (*p* > 0.05). Additionally, inappetence, nausea and vomiting, headache, and renal dysfunction were reported in one trial (Hu et al., 2021), and no statistical difference was identified in both combination treatment and control groups.

**TABLE 6:** Comparison of adverse drug events between the treatment group and control group.

Outcome measure	No. of trials	Samples			Statistical method	Effect estimate	*p*-value
		Total	Events/intervention	Events/control			
Adverse events rate	5	755	96/412	80/343	RR (random) 95%CI	1.72(0.44, 6.74)	0.44
Diarrhea	4	655	37/361	19/294	RR (random) 95%CI	2.43(0.20, 29.33)	0.49
Abnormal liver function	2	384	34/193	35/191	RR (fixed) 95%CI	0.97(0.64, 1.47)	0.88

### Sensitivity Analysis

Sensitivity analysis revealed a small change in the effect amount, and a significant difference in lung CT, clinical cure rate, rate of conversion to severe cases, symptom score of fever, cough reduction rate, symptom score of cough, WBC count, and CRP. Sensitivity analysis indicated that the above results were robust.

### Publication Bias

As the number of trials in any comparative outcome measure was less than ten, we did not assess the publication bias.

## Discussion

### Summary of Findings

In our study, it was found that Chinese herbal medicine honeysuckle could provide additional benefit for the clinical outcomes of COVID-19. This finding was consistent with similar studies (Ang et al., 2020; Xiong et al., 2020). Similar studies have shown that, compared with conventional therapy, Chinese herbal medicine had better effects and fewer adverse drug events (Ang et al., 2020; Xiong et al., 2020). Facing such a severe COVID-19 epidemic, Western countries should pay high attention to the curative effect of Chinese herbal medicine.

Honeysuckle is one of the most widely used traditional Chinese herbal medicines. It is used as an antiviral, immunomodulator, anti-inflammatory, hepatoprotectant, and nephroprotectant (Miao et al., 2019; Bai et al., 2020; Fang et al., 2020; Alekhya Sita et al., 2019). Honeysuckle is predicted to suppress SARS-CoV-2 replication (Lee et al., 2021). Honeysuckle extracts can inhibit the replication of influenza viruses H1N1, H3N2, and the oseltamivir-resistant mutant strain H1N1-H275Y (Li et al., 2020). Honeysuckle polysaccharides can regulate nonspecific immunity (Zhou et al., 2018) and inhibit the expression of inflammatory factors TNF-α and IL-1β (Bai et al., 2020). Neochlorogenic acid from Lonicera can prevent excessive macrophage-mediated responses associated with acute and chronic inflammatory disorders (Park et al., 2018). *Lonicera caerulea* L. polyphenols (LCPs) can alleviate LPS-induced liver injury by suppressing the nuclear translocation of NF-κB p65 and activation of the MAPK signaling pathway (Li et al., 2020). Luteolin is a pharmacologically active component normally found in honeysuckle, which exhibits antioxidant activity and nephroprotective activity (Alekhya Sita et al., 2019).

In our study, honeysuckle combined with conventional therapy was superior to conventional therapy alone in improving clinical symptoms, imaging, and laboratory indexes. Compared with conventional therapy alone, combination therapy of honeysuckle and conventional therapy could improve symptom score of fever, cough reduction rate, and symptom score of cough. We found that combination therapy could improve lung CT, increase WBC count, and reduce CRP level. This is related to the fact that honeysuckle can affect the immune response and production of inflammatory cytokines (Zhou et al., 2018; Bai et al., 2020). Immunopathological changes, including diminished lymphocytes and elevated cytokines, are important drivers of disease progression and death in coronavirus infections (Tang et al., 2020). Cytokine storm is uncontrolled overproduction of inflammation markers with elevated levels of IL-6, IL-1β, and TNF-α (Coperchini et al., 2020; Kempuraj et al., 2020). This leads to acute lung injury, acute respiratory distress syndrome (ARDS), and widespread tissue damage resulting in multiorgan failure and death (Ragab et al., 2020; Caricchio et al., 2021). In our study, we also found that combination therapy had improvements in clinical parameters including clinical cure rate and rate of conversion to severe cases.

Safety issues should be another concern when honeysuckle combined with conventional therapy is used for COVID-19 patients. In our study, all included trials reported adverse drug events. Compared with conventional therapy alone, combination therapy of honeysuckle and conventional therapy did not increase adverse drug events. This is related to the fact that honeysuckle exhibits hepatoprotective and nephroprotective activity (Alekhya Sita et al., 2019; Li et al., 2020).

### Strengths and Limitations

This study is to our knowledge the first systematic review and meta-analysis for the effectiveness and safety of honeysuckle combined with conventional therapy in adult patients with COVID-19. It could help to respond to the current public health emergency. Another advantage could be that only randomized studies are included. Our study was also performed in accordance with Cochrane Handbook and PRISMA checklist to draw quantitative conclusions with scientific and rigorous methods. In addition, we conducted a subgroup analysis and sensitivity analysis. It meant that our meta-analysis results were more robust.

However, our review had several limitations. Merger statistical analysis of some outcomes had unexplained heterogeneity. Most of the included trials had deficiencies in methodology design, including hidden allocation, blinding, and selective reporting. Publication bias was unclear. The drugs used in the control group were different. However, we did not perform subgroup analyses. The treatment duration ranged from 5 to 15 days. We also did not perform subgroup analyses according to treatment duration.

## Conclusion

In conclusion, honeysuckle combined with conventional therapy may be beneficial for the treatment of COVID-19 in improving clinical symptoms, lung CT, laboratory indicators, and clinical cure rate and reducing the rate of conversion to severe cases. Besides, combination therapy did not increase adverse drug events. However, considering the poor methodology of included trials, more high-quality trials are needed to evaluate the efficacy of honeysuckle in the treatment of COVID-19 in the future.
