# Supplementary material for: Add-On Effect of Honeysuckle in the Treatment of Coronavirus Disease 2019: A Systematic Review and Meta-Analysis
Source: Front Pharmacol. 2021 Sep 15;12:708636. doi: 10.3389/fphar.2021.708636 (PMC8479112; doi:10.3389/fphar.2021.708636)
Supplement: Supplementary file 1 [file Table1.DOCX]

**Supplementary Table 1:** Summary table of the studies included.

| **Study** | **Formulation** | **Source** | **Species, concentration** | **Quality control**  **reported?**  **(Y/N)** | **Chemical analysis**  **reported?**  **(Y/N)** |
| --- | --- | --- | --- | --- | --- |
| Ai. et al. (2020) | Pneumonia No.1 formula | Guangzhou Eighth People's Hospital | Lonicera japonica Thunb., 15 g  Hypericum perforatum L., 30 g  Artemisia annua L., 10 g  Astragalus mongholicus Bunge, 45 g  Cremastra appendiculata (D.Don) Makino, 20 g Scutellaria baicalensis Georgi, 10 g  Isatis tinctoria L., 10 g  Bupleurum chinense DC., 5 g  Peucedanum japonicum Thunb., 5 g  Fritillaria cirrhosa D.Don, 10 g  Fritillaria thunbergii Miq., 10 g  Prunus mume (Siebold) Siebold & Zucc., 30 g Scrophularia ningpoensis Hemsl., 10g  Smilax glabra Roxb. 30 g  Pseudostellaria heterophylla (Miq.) Pax, 15 g | Y- Prepared  according to Pharmacopoeia of the People's Republic of China | N |
| Ding. et al. (2020) | Qingfei Touxie Fuzheng recipe | General Hospital of the Central Theater of the Chinese people's Liberation Army | Lonicera japonica Thunb., 30 g,  Hypericum perforatum L., 15 g  Ephedra sinica Stapf, 6 g  Prunus armeniaca L., 10 g  Phragmites australis (Cav.) Trin. ex Steud., 30 g Coix lacryma-jobi L., 30 g  Reynoutria japonica Houtt., 15 g  Curcuma longa L., 10 g  Paeonia lactiflora Pall., 10 g  Pseudostellaria heterophylla (Miq.) Pax, 20 g Glycyrrhiza glabra L., 15 g | Y- Prepared  according to Pharmacopoeia of the People's Republic of China | N |
| Duan. et al. (2020) | Jinhua Qinggan granule | Juxiechang Beijing Pharmaceutical Co., Ltd. | Lonicera japonica Thunb.  Ephedra sinica Stapf  Prunus armeniaca L.  Scutellaria baicalensis Georgi  Hypericum perforatum L.  Fritillaria thunbergii Miq.  Anemarrhena asphodeloides Bunge  Arctium lappa L.  Artemisia annua L.  Mentha arvensis L.  Glycyrrhiza glabra L. | Y- Prepared according to National Drug Standards of China Food and Drug Administration (Z20160001) | N |
| Fu. et al. (2020) | Toujie Quwen granule | Guangdong Yifang Pharmaceutical Co., Ltd. | Lonicera japonica Thunb., 15 g  Hypericum perforatum L., 30 g  Artemisia annua L., 10 g  Astragalus mongholicus Bunge, 45 g  Cremastra appendiculata (D.Don) Makino, 20 g Scutellaria baicalensis Georgi, 10 g  Isatis tinctoria L., 10 g  Bupleurum chinense DC., 5 g  Peucedanum japonicum Thunb., 5 g  Fritillaria cirrhosa D.Don, 10 g  Fritillaria thunbergii Miq., 10 g  Prunus mume (Siebold) Siebold & Zucc., 30 g Scrophularia ningpoensis Hemsl., 10g  Smilax glabra Roxb. 30 g  Pseudostellaria heterophylla (Miq.) Pax, 15 g | Y- Prepared  according to Pharmacopoeia of the People's Republic of China | N |
| Fu. et al. (2020) | Toujie Quwen granule | Guangdong Yifang Pharmaceutical Co., Ltd. | Lonicera japonica Thunb., 15 g  Hypericum perforatum L., 30 g  Artemisia annua L., 10 g  Astragalus mongholicus Bunge, 45 g  Cremastra appendiculata (D.Don) Makino, 20 g Scutellaria baicalensis Georgi, 10 g  Isatis tinctoria L., 10 g  Bupleurum chinense DC., 5 g  Peucedanum japonicum Thunb., 5 g  Fritillaria cirrhosa D.Don, 10 g  Fritillaria thunbergii Miq., 10 g  Prunus mume (Siebold) Siebold & Zucc., 30 g Scrophularia ningpoensis Hemsl., 10g  Smilax glabra Roxb. 30 g  Pseudostellaria heterophylla (Miq.) Pax, 15 g | Y- Prepared  according to Pharmacopoeia of the People's Republic of China | N |
| Hu. et al. (2020) | Jinyinhua oral liquid | Hubei Zhenao Jinyinhua Pharmaceutical Co., Ltd. | Lonicera japonica Thunb., 1.8g | Y - Prepared  according to Pharmacopoeia of the People's Republic of China | N |
| Hu. et al. (2020) | Lianhua Qingwen capsule | Beijing Yiling Pharmaceutical Co., Ltd. | Lonicera japonica Thunb.  Hypericum perforatum L.  Ephedra sinica Stapf  Prunus sibirica L.  Isatis tinctoria L.  Dryopteris crassirhizoma Nakai  Houttuynia cordata Thunb.  Ageratum conyzoides L.  Rheum palmatum L.  Rhodiola rosea L.  Mentha × piperita L.  Glycyrrhiza glabra L. | Y - Prepared according to National Drug Standards of China Food and Drug Administration (Z20040063) | N |
| Yu. et al. (2020) | Lianhua Qingwen granule | Beijing Yiling Pharmaceutical Co., Ltd. | Lonicera japonica Thunb.  Hypericum perforatum L.  Ephedra sinica Stapf  Prunus sibirica L.  Isatis tinctoria L.  Dryopteris crassirhizoma Nakai  Houttuynia cordata Thunb.  Ageratum conyzoides L.  Rheum palmatum L.  Rhodiola rosea L.  Mentha × piperita L.  Glycyrrhiza glabra L. | Y - Prepared according to National Drug Standards of China Food and Drug Administration (Z20100040) | N |
| Zhang. et al. (2020) | Jinyinhua oral liquid | Hubei Zhenao Jinyinhua Pharmaceutical Co., Ltd. | Lonicera japonica Thunb., 1.8g | Y - Prepared  according to Pharmacopoeia of the People's Republic of China | N |
